# Recent trends and advances in polyindole-based nanocomposites as potential antimicrobial agents: a mini review

**DOI:** 10.1039/d1ra09317g

**Published:** 2022-03-15

**Authors:** Hareesh Pradeep, Bindu M., Shwetha Suresh, Anjitha Thadathil, Pradeepan Periyat

**Affiliations:** Department of Chemistry, University of Calicut Kerala India-673635; Department of Environmental Studies, Kannur University Kerala India pperiyat@uoc.ac.in pperiyat@kannuruniv.ac.in

## Abstract

Infections caused by multi-drug resistant microbes are a big challenge to the medical field and it necessitates the need for new biomedical agents that can act as potential candidates against these pathogens. Several polyindole based nanocomposites were found to exhibit the ability to release reactive oxygen species (ROS) and hence they show excellent antimicrobial properties. The features of polyindole can be fine-tuned to make them potential alternatives to antibiotics and antifungal medicines. This review clearly portrays the antimicrobial properties of polyindole based nanocomposites, reported so far for biomedical applications. This review will give a clear insight into the scope and possibilities for further research on the biomedical applications of polyindole based nanocomposites.

## Abstract 2

This review clearly portrays the antimicrobial properties of polyindole based nanocomposites, reported so far for antimicrobial applications and it gives clear insight into the scope and possibilities for further research on the biomedical application of polyindole based nanocomposites.

## Graphical abstract

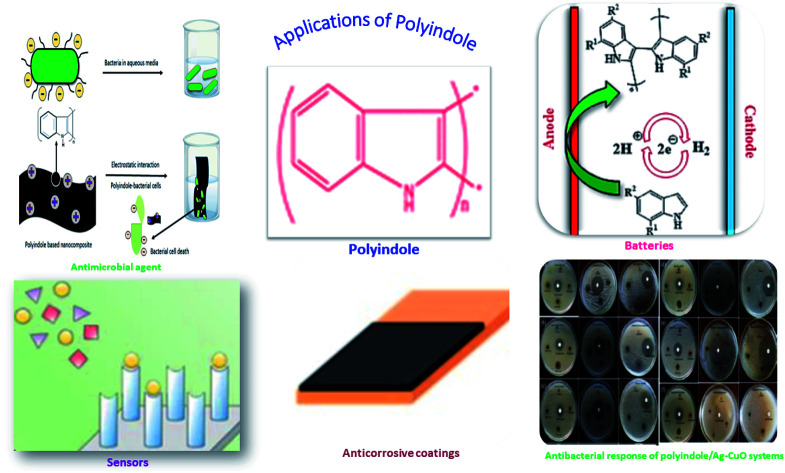

## 1. Introduction

Microorganism induced resistance, based on the built-in abilities to nullify the activity of current antibiotics has been considered very crucial in regards to public health, especially in a scenario of the alarming increase in untreatable bacterial infections and scarcity in the production of new antibiotics.^1–3^ One alternative to address the impact of this issue is prevention, *i.e.*, hampering the growth and development or simply preventing their adhesion.^4–6^ In this regard, the development of novel antibacterial materials to avoid the usage of antibiotics becomes an excellent approach. The replacement of conventional antibiotics by nanocomposites presents important advantages to deactivate new strategies of intrinsic resistance developed by multidrug-or even pan-drug-resistant microorganisms.^7,8^

The exciting properties of nanotechnology have led to the development of antimicrobial nanomaterials in recent years. Nanomaterials can be used as an alternative to antibiotics, because of the ease in fine tuning of their properties such as particle size, crystal defect and morphology.^9^ Understanding the mechanism of antibacterial activity of nanomaterials is important in controlling the *in vivo* dosage.^10^ The ability of the material to produce reactive oxygen species (ROS) is of precise attention in regards to toxicity, owing to the oxidation of various cellular constituents by oxygen centered reactive species. ROS may comprise superoxide anions (O_2_˙^−^), hydroxyl radicals (OH˙), singlet oxygen and secondary oxygen centered species such as H_2_O_2_ (formed by the disproportionation of O_2_˙^−^) which then converted to OH˙ and singlet oxygen.^11–13^ Excellent literature reports are available based on the antibacterial activity of nano ZnO, TiO_2_, MgO, CuO, ZnO/TiO_2_ hybrids and Ag_3_PO_4_.^14–18^ It has been shown that, the ROS generated by nanomaterials can be used to treat cancer cells^11,19–21^ and ROS generation strongly depends upon the shape, size, surface area, charge and heterostructure of the nanomaterials.

**Fig. 1:**
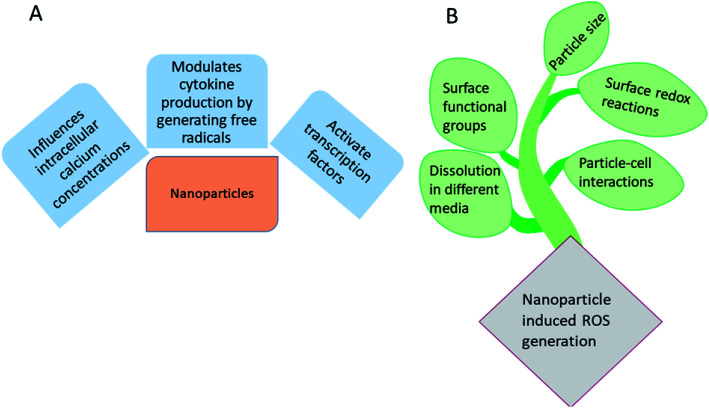
(A) Influence of metallic and metallic oxide nanoparticles on the living systems and (B) major factors involved in the nanomaterials induced ROS generation.

Fig. 1(A) represents the influence of metallic and metallic oxide nanoparticles on the living systems and Fig. 1(B) represents the factors influencing the nanomaterials induced ROS generation. As the size of the materials becomes nano dimensions, there may be structural defects, owing to which alteration in the surface properties occurs. Electron donor or acceptor, then reacts with oxygen, leading to the formation of superoxide anions (O_2_˙^−^), which further undergoes Fenton type reactions^22–24^ to generate additional ROS. According to Fenton mechanism, the metal or metallic oxide nanoparticles react with H_2_O_2_ to form OH˙ and oxidized metal ion. There is one more mechanism, *i.e.* Haber Weiss mechanism, in which generation of OH˙ *via* the reaction between H_2_O_2_ and oxidized metal ions.^25–28^

**Fig. 2:**
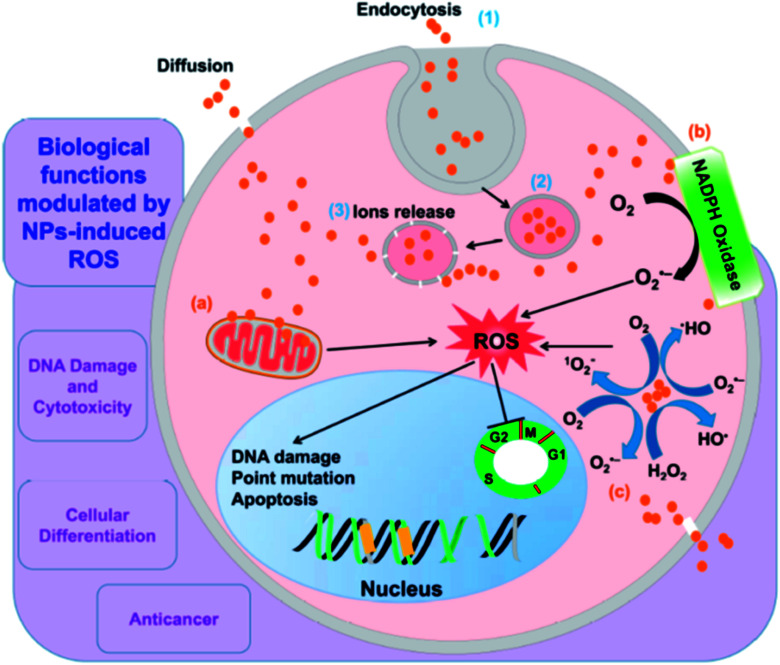
A schematic representation of mechanism of nanoparticle induced ROS generation. Reprinted with permission from ref. 29; copyright © MDPI.

Fig. 2 shows a schematic representation of the mechanism of nanoparticle induced ROS generation. Various steps involved are: (1) endocytosis (2) generation of endocytotic vesicles (3) release of nanoparticles from vesicles into the cell. The nanoparticle may then interact with mitochondria and NADPH oxidase, leading to the formation of ROS, owing to which DNA damage, cell cycle termination and alteration in apoptosis occurs.^29^

It has been reported that H_2_O_2_ can induce oxidative stress on living cells by forming ROS intracellularly.^30,31^ The intracellular ROS formation may happen either by some metabolic process (endogenous) or by other entities such as nanoparticles (exogenous).^32–34^ The ROS facilitated antibacterial activity has been found to be pro-inflammatory.^35–37^ Investigation of the antioxidant features of nanomaterials towards macrophages is of particular attention, owing to the fact that macrophage targeting may be employed to deliver anti-inflammatory drugs at the site of inflammation.

It has been reported that the inflammation (swelling at a particular area, pain and redness due to some injury or infection) is enhanced by some mechanism including ROS generation in macrophages.^38,39^ Studies reveal that the size of nanoparticles plays a crucial role in their uptake by macrophages.^40^ Materials which exhibit both antibacterial and anti-inflammatory properties simultaneously have potential for a variety of biomedical applications.^41–45^

Photodynamic therapy (PDT) is a method of bacterial inactivation through oxidative stress by photosensitization. The photosensitizer absorbs specific wavelength of light, usually from laser sources, followed by visible light irradiation generates ROS.^46,47^ Uncontrolled formation of ROS leading to cell damage and cell death. The photosensitizer is either put into the blood stream *via* veins or applied directly on skin. After a certain period of time, drug is absorbed by the harmful cells. Upon irradiation to the area to be treated, the drug reacts and kills the cells. The time gap between the drug is given and light irradiation is known as drug to light interval.^48^

**Scheme 1:**
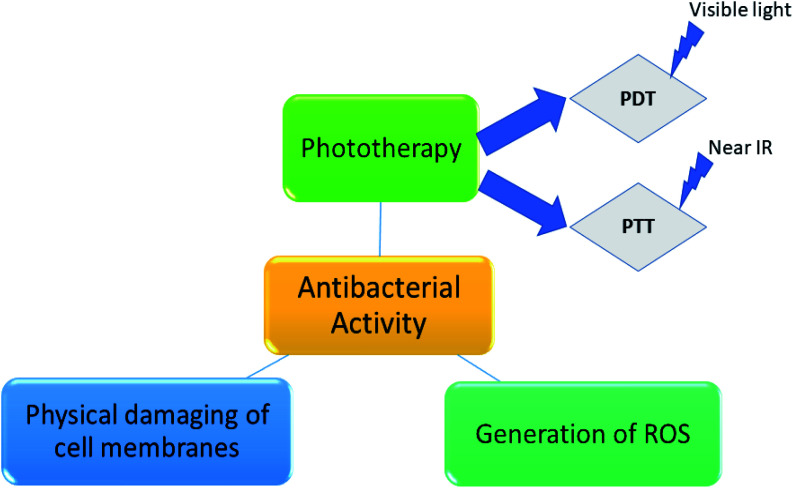
Different pathways of antibacterial activity.

As a replacement of PDT by photothermal therapy (PTT), also requires photosensitizer which converts light energy into heat, owing to which cell impairment occurs. Generation of heat leads to aggregation and denaturation of the proteins, causes cell death. Here, the irradiation is done by near IR light.^49,50^Scheme 1 shows different mechanistic pathway of antibacterial activity.

Nowadays, polymer nanocomposites have extensively been employed for antibacterial, tissue engineering, cancer therapy, medical imaging, drug delivery and dental applications. Polymer nanocomposites consist of a macromolecular matrix, in which nano fillers are embedded. Polymers are excellent hosting materials to fabricate composites, because of the easiness in tailoring their characteristics, to obtain a system, having good processability and durability. Addition of nanofillers to such matrices generates a material, with desired and fine-tuned properties than their counterparts.^50–55^ Polylactide, poly-glycolide and polycaprolactone are biodegradable and biocompatible polymers.^56–58^ Polymer nanocomposites, based on chitosan, poly(*N*-vinyl-2-pyrrolidone) (PVP), polyvinyl alcohol (PVA) and polyvinyl chloride (PVC) have been investigated for antimicrobial properties' evaluation and Table 1 lists a concise literature report of some commonly employed polymer nanocomposites as antimicrobial medicine.

**Table 1:** Commonly employed polymer nanocomposites as antimicrobial medicine^a^

Polymer nanocomposites	Compositions	Species	CFU mL^−1^	Antibacterial activity performances	Biocompatibility tests	Ref.
Chitosan/GO/iron oxide	0.1 wt% GO/iron oxide	*S. aureus* (Gram positive)	1 × 10^5^	DIZ ∼15 mm	Causing concentration dependent hemolysis of human red blood cells	63
		*E. coli* (Gram negative)	1 × 10^6^	DIZ ∼15 mm		
Chitosan/GO/ZnO	NG	*S. aureus*	1 × 10^6^	MIC: 0.1 μg mL^−1^	—	64
		*E. coli*	1 × 10^6^	MIC: 0.1 μg mL^−1^		
Chitosan/GO/TiO_2_	Chitosan : GO : TiO_2_ (1 : 20 : 4)	*B. subtilis* (Gram positive)	1 × 10^8^	With 40 μg mL^−1^ of the material, the OD_600_ of *B. subtilis* drop from 0.79 to 0.33 for 12 h	Not causing cytotoxicity against mammalian somatic cells and plant cells	65
PVA/GO	0.1 wt% GO	*S. aureus*	2 × 10^5^	No obvious (24 h)	—	66
		*E. coli*	2 × 10^4^			
PVA/GO/AgNPs	0.01 wt% GO, 10 wt% PVA, 3.9 mM AgNO_3_	*S. aureus*	1 × 10^6^	BR: 100% (3 h)	—	67
		*E. coli*	1 × 10^6^	BR: 100% (3 h)		
PLA/GO–ZnO	0.2 wt% GO–ZnO	*S. aureus*	—	BR: 83% (24 h no light), 99% (24 h with light)	—	68
		*E. coli*	—	BR: 52% (24 h no light), 98% (24 h with light)		
PAM/rGO/Ag	1 wt% PAM/rGO	*S. aureus*	—	DIZ: 47 mm	—	69
		*Pseudomonas* (Gram negative)	—	DIZ: 45 mm		

a BR: bactericidal rate, DIZ: diameter of inhibitory zone, MIC: minimum inhibitory concentration, CFU: colony forming units.

## 2. Conducting polymer nanocomposites as biomedical agents

Conducting polymers (CPs) are a specific category of synthetic polymers with exceptional electrical and optical characteristics, which involve conjugated chains with alternating single and double bonds.^59,60^ Polyacetylene (PA), polythiophene (PT), poly[3,4-(ethylenedioxy)thiophene] (PEDOT), polypyrrole (PPy), polyindole (PIN), polyphenylene and polyaniline (PANi) are some examples of the most extensively used CPs in biomedical area.^61^ CPs have demonstrated promising candidates for numerous biomedical applications due to their biocompatibility, gifted response to electrical fields, high electrical conductivity, low-toxicity, good environmental stability, and nanostructured morphology.^62^ Recently, conducting polymers are widely used as antimicrobial and antifungal agents in various sectors such as bio-medical field, food industry, coating industry *etc.* The tendency for CPs to have low processability and not to be degradable, which can potentially be overcome by the synthesis of degradable CPs that are solution processable, and fabrication of CP blends and nanocomposites with various (bio) polymers and nanomaterials, respectively. Table 2 give a complete literature analysis of conducting polymer nanocomposite materials reported so far as antimicrobial medicine.

**Table 2:** Conducting polymer nanocomposite materials as antimicrobial medicine^a^

Conducting nanocomposites	Species	CFU mL^−1^	Antibacterial activity performances	Biocompatibility tests	Ref.
PPy–Pd	*S. aureus* (Gram positive)	—	MIC: 5.78 mg mL^−1^	—	70
			MBC: 23.12 mg mL^−1^		
PPY–Zn@CuO	*E. coli* (Gram negative)	—	MIC: 0.078 mg mL^−1^	PPY–Zn@CuO pertaining to minimal cytotoxicity	71
	*S. aureus*	—	MIC: 0.156 mg mL^−1^		
PANI–Zn@CuO	*E. coli*		MIC: 0.144 mg mL^−1^	PANI–Zn@CuO and PPY–Zn@CuO pertaining to minimal cytotoxicity	71
	*S. aureus*		MIC: 0.144 mg mL^−1^		
PPy–NT Ag-NP	*E. coli*	—	MIC: 0.078 mg mL^−1^	—	72
	*S. aureus*	—	MIC: 0.15625 mg mL^−1^		
polyaniline/Pt–Pd	*Staphylococcus* sp (Gram positive)		MIC: 25 mg mL^−1^	—	73
			MBC: 150 mg mL^−1^		
AuNP–PTh	*E. coli* MTCC 433	1 × 10^6^	MBC: 112 μM	No harmful influence of AuNP–PTh treatment for various time periods (24 and 48 h)	74
	*L. monocytogenes* Scott A (Gram positive)	1 × 10^6^	MBC: 112 μM		
Cu–PANI	*E. coli*	1 × 10^6^	—	—	75
	*S. aureus*	1 × 10^6^			

a MBC: minimum bactericidal concentration.

### 2.1. Polyindole (PIN)

Owing to the unique physical and electrochemical properties, polyindole (PIN) has gained marvellous consideration of the researchers, in the past couple of years. They belong to the fused ring compound family, which possesses a benzene and a pyrrole ring; so polyindole have the features of poly(*para*-phenylene) and polypyrrole^76^ as well. Several studies on polyindole revealed that they could be used as promising candidates for applications such as supercapacitors, batteries, electrochromic devices, sensors, electrocatalysis, catalysis, and anticorrosion.^77–83^ Graphene and silver nanoparticles loaded polyindole have been subjected to electro activity studies and found that such system can be used as an electrode material for various applications.^84^

**Fig. 3:**
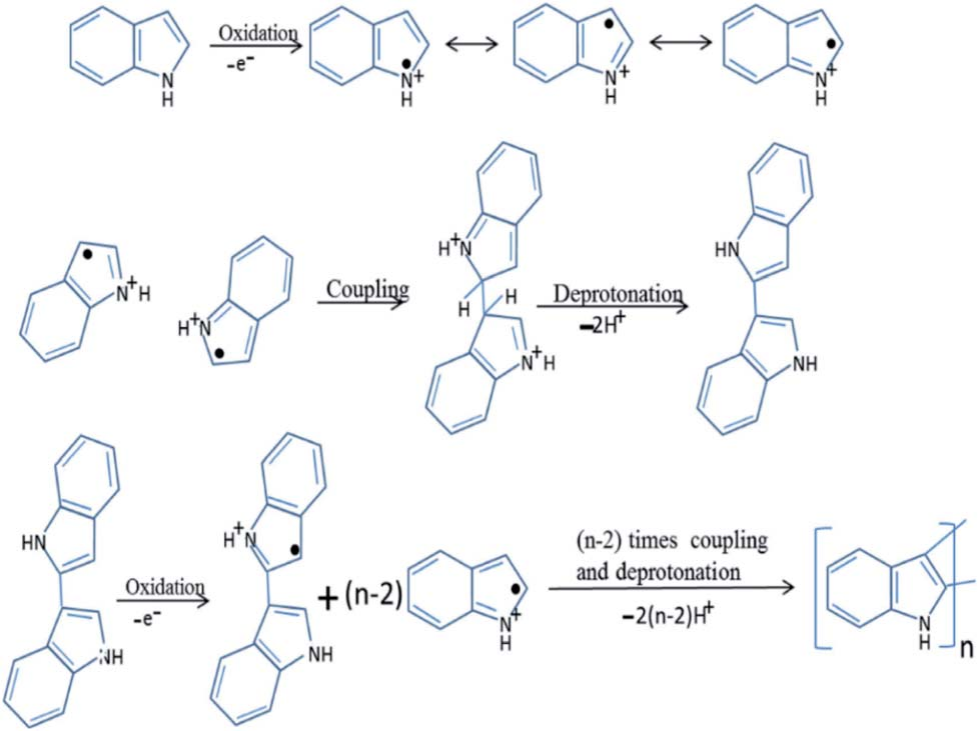
Mechanism of chemical polymerization of PIN. Reprinted with permission from ref. 88; copyright ©Elsevier.

In the early 1976, initial studies have begun on the development of chemical polymerization methods to synthesize polyindole from indole.^85^ In 1982, Tourillon and Garnier synthesized conducting polyindole by employing electrochemical methods.^86^ Compared to polyaniline and polypyrrole, polyindole exhibit high thermal stability (crucial for sterilization, *e.g.*; in an autoclave), excellent oxidation–reduction activity (redox activity), chargeable electrical conductivity, slow rate of degradation and good blending properties.^87^ Because of its exceptional advantages in various domains, many scientists have done healthy research on polyindole and their derivatives in terms of their synthesis, properties, structure and applications. Two strategies have been employed for the synthesis PIN from indole monomers, chemical oxidative polymerization and electrochemical polymerization.^88^ We can precisely control the morphology of PIN formed such as nanowires, nanorods, nano- and micro-fibers, nano- and micro-spheres, and nanobelts.^78^ The chemical oxidative polymerization technique has been employed for the large scale production of PIN.^89^ The mechanism involves the formation of radical cations, by the oxidation of indole monomer and these indole radical cations couple together at 2 and 3 position. The deprotonation of the coupled species results in the formation of a dimer, which again undergoes oxidation, coupling and deprotonation, results in the formation of a trimer and the chain propagates to form the final product as polyindole. The mechanism has been depicted as Fig. 3.

**Fig. 4:**
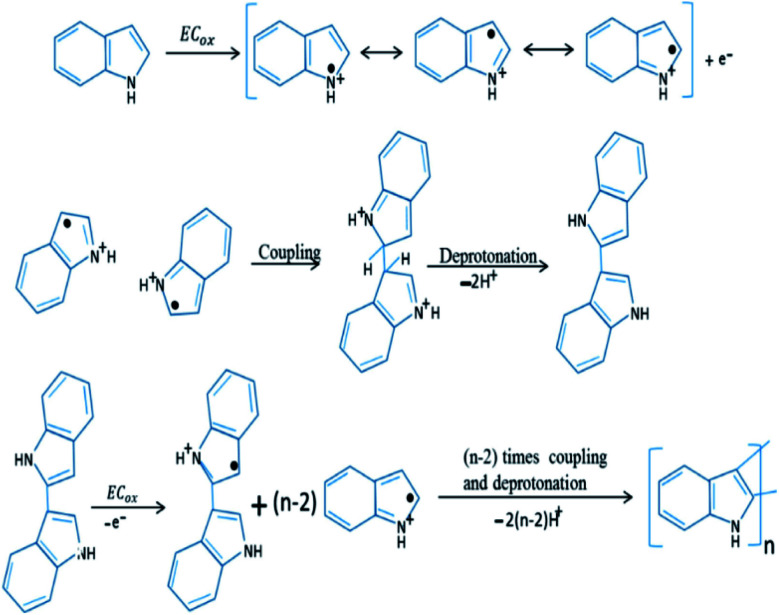
Electrochemical polymerization mechanism of PIN formation. Reprinted with permission from ref. 88; copyright © Elsevier.

Unlike the chemical polymerization methods, electropolymerization produces PIN directly on a target electrode substrate in a three electrode system. A binder-free electrode has been achieved by using an organic or non-organic electrolyte and dopant material.^89^ Mechanism of the electrochemical polymerization of PIN has been shown as Fig. 4. The coupling position of indole moieties during the polymerisation strongly depends upon the nature of the solution and electrolyte used.^90^

Polyindole based nanocomposites exhibit outstanding antimicrobial properties because of its promising capability to generate ROS, it can effectively inhibit microbial growth. Although many metal and metal oxide nanoparticles offer excellent antimicrobial activity, their cytotoxicity and safety concerns still exist as a challenge.^91^ Polyindole based nanocomposites have been found to exhibit enhanced antimicrobial activity than its partners due to the mutual synergistic enhancement of their properties.^92^ Also, they have less cytotoxic effects on human bodies. Hence, polyindole nanocomposites can be substituted as a potential alternative for antibiotics and can act as an effective biomedical agent.

### 2.2. Antimicrobial features of polyindole based nanocomposites

**Fig. 5:**
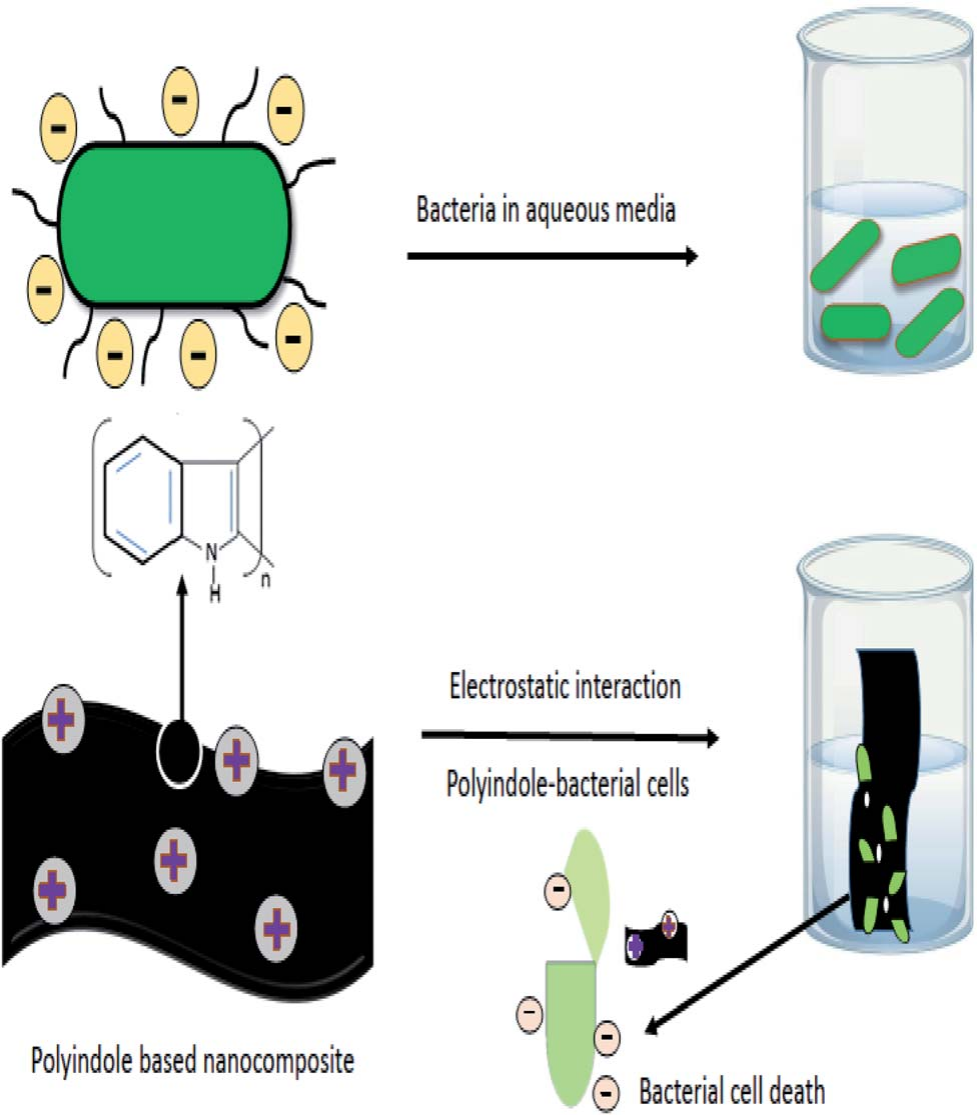
Schematic representation of electrostatic interactions involved in the mechanism of antibacterial activity of polyindole nanocomposites.

The oxidative polymerization of indole moieties produces positive charges at fixed intervals of monomers, along the polymeric chain of polyindole. This cationic nature is responsible for the antibacterial activity of the resulting PIN. The positive charge of polyindole chains electrostatically interacts with the negatively charged surfaces of bacterial cell wall, irreversibly interrupting the membrane structure of the bacteria, leading to penetration through the cells, and efficiently hindering the protein activity.^87^ Owing to the interaction with the charged surfaces and the diffusion of reactive species into the cell wall, cell death occurs by the leakage of vital components from the cells. A schematic representation of the electrostatic interactions and the step of cell death has been shown as Fig. 5.

The incorporation of nanomaterial into polyindole matrices enhance the performance against bacterial growth, proliferation, and the following cell death owing to the synergistic interaction of the components. With this goal, the polyindole has been combined with different fillers such as Ag, Ag–CuO, Ag–ZnO, Ag/CeO_2_, Ag/Co_3_O_4_, graphene, ZrO_2_, TiO_2_, and NiO/ZnO nanoparticles. In the following section, strategies for the optimization of polyindole-based nanocomposites as antibacterial agents have been discussed.

#### 2.2.1. Polyindole/Ag based nanocomposites

Ag nanoparticles constitute a class of biologically important metallic nano particles, which can be employed as an antimicrobial agent.^93^ It has been reported that, the incorporation of Ag nanoparticles into polymeric matrices, impart antimicrobial properties to the latter or enhance it.^94^ By combining the advantages of both the partners *i.e.*, Ag nanoparticles and the polymers, such as strong antimicrobial effect of nano Ag, unique features of polymers such as exceptional structural consistency, various morphologies and architecture and varying chemical compositions, leads to the development of a composite systems with improved properties.^95–97^

The antimicrobial activity of Ag and its ionic form is due to the binding of metallic ions to certain bio-macromolecular components. It has been reported that the cationic Ag targets binds to negatively charged components of the proteins and nucleic acid, leads to structural deformations in cell membrane and nucleic acids.^98–100^ Ag ions can also interact with electron rich functional groups such as thiols, hydroxyls, imidazoles, phosphates, indoles and amines.^100^ The binding of Ag ions to DNA, block transcription whereas those binds to cell surface inhibits bacterial respiration and ATP (adenosine triphosphate) synthesis and Ag ions have the potential to block the respiratory chain of microorganism in the cytochrome oxidase and NADH-succinate dehydrogenase region.^10^ Various mechanisms have been suggested to describe the antimicrobial activity of Ag nanoparticles. They are (1) slow release of Ag ions followed by suppression of ATP production and replication of DNA (2) cell membrane damaging directly (3) Production of ROS.^101^ Electron spin resonance (ESR) studies have been used to confirm the ROS generation.

**Fig. 6:**
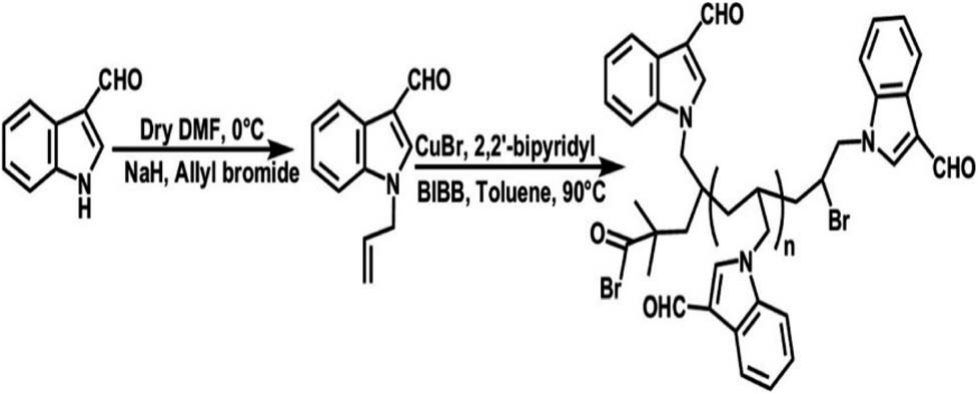
Synthesis strategy of poly(1-allylindole-3 carbaldehyde). Reprinted with permission from ref. 92; copyright © Wiley.

Many polyindole derivatives have been prepared till now, in regards to the antimicrobial activity of indole monomer, some them shows fungicidal activity. In an interesting work, 1-allylindole-3 carbaldehyde (AIC) was used as the monomer and polymerization was carried out by atom transfer radical polymerization (ATRP) strategy, to form a polyindole derivative.^92^ The synthesis strategy has been depicted as Fig. 6.

Ag nanoparticles were prepared by using solutions of AgNO_3_ and NaBH_4_ as precursors. Addition of AgNO_3_ to NaBH_4_, causes the reduction of AgNO_3_ to Ag nanoparticles. A thin layer of borohydride anion got absorbed on the surface of nanoparticles, prevents their agglomeration. Ag nanoparticles synthesized were then introduced into the polymer matrix by ultrasonic-assisted method.^93^

Antibacterial studies were carried out against human pathogenic bacteria, by two different methods, disc diffusion method and broth dilution method.^102^ The results obtained from both the methods were similar, and observed that the monomer AIC doesn't exhibit any antibacterial properties at all, but the polymer PAIC [poly(1-allylindole-3 carbaldehyde) and the nanocomposite (PAICN) does. Table 3 shows the response of different systems towards pathogens.

**Table 3:** The response of AIC, PAIC and PAICN towards pathogens. Reprinted with permission from ref. 92; copyright © Wiley^a^

Sample	Gram positive bacteria		Gram negative bacteria		
	*E. faecalis*	*S. aureus*	*E. coli*	*P. mirabilis*	*K. pneumoniae*
**Disc diffusion data**					
AIC	—	—	—	—	—
PAIC	—	+	—	++	+++
PAICN	++	—	—	—	+

**Minimum inhibitory concentration (MIC, in μg mL** ^**−1**^ **) against pathogens, determined after one day of incubation**					
AIC	—	—	—	—	—
PAIC	—	>50	—	50	40
PAICN	35	—	—	—	>50

a —, no antibacterial activity; +, less than 7 mm; ++, 8–15 mm; +++, more than 15 mm.

The association of aldehyde group of PAIC with unprotonated amines on the outer layer of bacterial cells is responsible for its antibacterial activity.^103^ An insignificant activity has been observed against *S. aureus* and *E. faecalis*. They exist as mucoid strains and their cells are being enclosed by a slime coating. The non-mucoid strains are affected more quickly compared to mucoid strains. PAICN shows activity against *E. faecalis*, with a minimum inhibitory concentration of 35 μgmL^−1^, owing to the slow release of Ag nanoparticles from the matrix. But, no activity was observed against *S. aureus*, *P. mirabilis* and *K. pneumoniae*, since the aldehyde group can't bind with amino acids on the cell surface, they are being participated in stabilizing Ag nanoparticles, owing to the affinity of oxygen atom towards metals.^104^

Antimicrobial features of ZnO nanoparticles are well known, which makes them suitable for agriculture and anticancer treatment.^105–109^ Oxidative stress mechanism involving ZnO nanoparticles against *E. coli*, have been well reported.^102^ For bulk ZnO, external generation of H_2_O_2_, is the reason for antibacterial activity. Being amphoteric, ZnO reacts with both acidic and alkaline medium, to generate Zn^2+^ ions. The free Zn^2+^ ions may then combine with proteins and carbohydrates, ceases the vital functions of bacteria.^108^

In view of the biological characteristics of ZnO nanoparticles, researchers are involved in the fabrication of hybrid materials, in combination with Ag nanoparticles, to achieve excellent antibacterial properties. Ag–ZnO nanocomposites can be used as an effective antimicrobial agent against a number of pathogenic bacteria. A recent study assessed the bactericidal effect of Ag–ZnO nanocomposites with *S. aureus* (Gram-positive) and GFP (green fluorescent protein, Gram-negative recombinant) expressing antibiotic resistant *E. coli*.^109^ By introducing these metal nanocomposites on a polymer matrix, its durability can be improved as well as the cytotoxic effects can be minimized.

In an interesting work, polyindole/Ag–ZnO nanocomposites were synthesized *via* chemical oxidation and co-precipitation methods and their antibacterial activities were explored.^110^ The antibacterial efficiency was assessed in terms of concentration of both AgNO_3_ and polyindole. Formation of the nanocomposites have been confirmed by using XRD, FTIR, SEM-EDAX and TEM. The selected bacterial strains for this study were *E. coli*, *P. mirabilis*, *E. faecalis*, *B. subtilis*, *S. epidermidis*, and *S. aureus*. The order of increasing bactericidal efficiency in terms of inhibition zone against the microbes follows the order, *B. subtilis* > *E. coli* > *E. faecalis* > *S. aureus* > *P. mirabilis* > *S. epidermidis.* The data of average zones of inhibition have been presented in Table 4.

**Table 4:** Average inhibition zone for polyindole/Ag–ZnO nanocomposites, nano ZnO, nano Ag and polyindole (in mm). Reprinted with permission from ref. 110; copyright © Elsevier

Microbes	Gram staining	ZA1	ZA2	ZA3	ZA4	ZA5	ZnO	Ag	Pin
*E. coli*	Gram negative	18	17	16	18	17	5	7	5
*P. mirabilis*		13	13	13	13	13	4	8	—
*E. faecalis*	Gram positive	16	14	13	14	13	6	16	4
*B. subtilis*		23	20	20	20	20	4	11	—
*S. epidermidis*		12	11	11	13	13	4	7	5
*S. aureus*		15	13	12	12	13	3	12	4

It has been shown that the polyindole/Ag–ZnO nanocomposites possess good bactericidal efficiency than their constituents. However, the concentration of AgNO_3_ did not play any crucial role in enhancing the overall antibacterial effect. When the nanoparticles are incorporated into the polymer matrix, their exposure gets restricted, leading to a reduction in the cytotoxic effects towards healthy mammalian cells. Hence, the use of polyindole/Ag–ZnO nanocomposites promote a biocompatible, non-cytotoxic and thereby a safe approach in treating bacterial infections.

Elemental Cu and its compounds have been identified as antimicrobial agents by US environmental protection agency (EPA).^111^ Both Cu (+1) and Cu (+2) oxides in the nano-dimension exhibit excellent antimicrobial characteristics against many pathogens. The antimicrobial properties strongly depend upon their particle size, morphology and dissolution of copper ions in different media. The redox cycling between Cu^+^ and Cu^2+^ generates superoxide species, causes the degradation of biomolecules.^112^

Ag/CuO nanocomposites have been subjected to antimicrobial activity evaluation against Gram positive microbe *Streptococcus pneumoniae*.^113^ High surface-volume ratio of the nanoparticles makes their contact with the microbial cell surfaces, leading to cease the cellular functions.

**Fig. 7:**
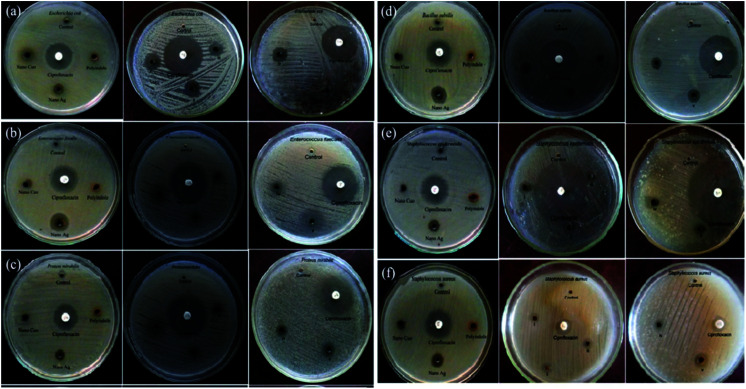
Antibacterial response of polyindole/Ag–CuO systems and its constituents against different bacterial strains. Reprinted with permission from ref. 114; copyright ©Taylor and Francis Ltd.

Polyindole/Ag–CuO systems have been developed *via* a reflux strategy and their antimicrobial efficiency were assessed by well diffusion method.^114^ While preparing the nanocomposites, the concentration of both polyindole and AgNO_3_ has been varied and those of CuO kept constant. The structural characterisations of the prepared nanocomposites were done by FTIR, XRD and SEM analysis. The selected bacterial strains for this study were *E. coli*, *P. mirabilis*, *E. faecalis*, *B. subtilis*, *S. epidermidis*, and *S. aureus.* The antibacterial activity has been compared with the standard ciprofloxacin.^73^Fig. 7 represent the antibacterial responses of polyindole/Ag–CuO systems against the pathogens.

**Fig. 8:**
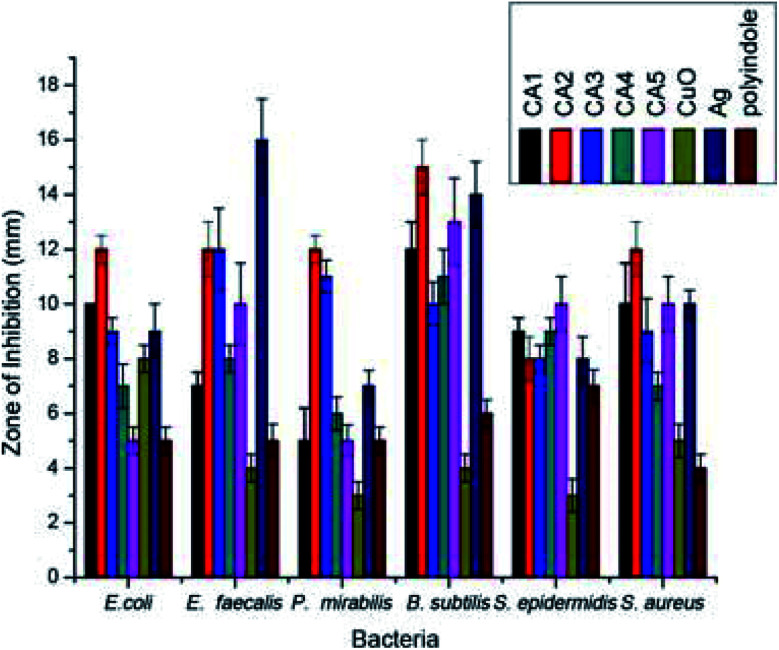
A Comparison of antibacterial activity of polyindole/Ag–CuO systems. Reprinted with permission from ref. 114; copyright © Taylor and Francis Ltd.

It has been observed that the polyindole/Ag–CuO nanocomposites exhibit ∼50% activity in comparison with the reference antibiotic, against the pathogen. The antibacterial activity of the nanocomposites has been compared and shown as Fig. 8. The inhibition zone diameter found with nano CuO, Ag and polyindole were 6, 11 and 5 mm respectively. But for polyindole/Ag–CuO nanocomposites, an average zone diameter of 12 mm has been observed.

Since the nanocomposites can easily interact with the bacterial cell wall, the released nanoparticles can effectively penetrate into the bacterial cell causing toxicity to the cells.^99^ Electrostatic interaction between the nanocomposites and cell wall of bacteria eventually leads to cell death.^115^ The results unveil the possibilities of exploring polyindole/Ag–CuO nanocomposites as an effective antimicrobial agent against pathogenic bacteria.

Cerium and cerium oxide-based nanomaterials have gained considerable attention as effective antibacterial agent against many pathogens, owing to the ROS induced by reversible conversion of oxidation state between Ce (+3) and Ce (+4).^116^ Literature reports are available based on the incorporation of Ce and CeO_2_ into many polymeric matrices for antibacterial applications.^117,118^

The antibacterial properties of Ag/CeO_2_ nanocomposites were comprehensively discussed in a recent article.^119^ The antibacterial activity of the nanocomposites has been assessed against *S. aureus* and *P. aeruginosa*, Gram-positive and Gram-negative bacteria respectively. For both the bacterial strains, the MIC upon treatment of Ag/CeO_2_ nanocomposites were observed to be 3.125 μg mL^−1^ and 6.25 μg mL^−1^ respectively.

Polyindole based Ag doped CeO_2_ nanocomposites were explored for its antibacterial properties.^120^ Amorphous nature of the nanocomposites has been confirmed from the XRD results. The porous polyindole, spherical Ag and CeO_2_ nanoparticles were identified from the SEM and TEM investigations. Polyindole/Ag–CeO_2_ systems exhibited better antibacterial properties than their constituents. The average zone of inhibition against various bacterial strains have been presented as Table 5.

**Table 5:** Inhibition zone (average, in mm) of nano Ag, nano CeO_2_, polyindole and polyindole/Ag–CeO_2_ nanocomposites. Reprinted with permission from ref. 120; copyright © Elsevier

S. no.	Bacteria	Gram staining	Nano CeO_2_	Nano Ag	Polyindole	Nanocomposites		
						CM1	CM2	CM3
1	*B. subtilis*	Gram positive	8.1	7.2	6.3	10.1	12.2	14.3
2	*S. aureus*		9.2	6.3	8.2	10.3	8.2	9.1
3	*S. pneumoniae*		4.2	6.2	2.3	7.2	8.3	10.2
4	*E. coli*	Gram negative	2.3	7.3	8.3	14.2	12.1	11.3
5	*P. vulgaris*		8.2	6.3	4.2	7.3	8.2	10.2
6	*K. pneumoniae*		12.2	6.2	10.3	10.2	9.3	10.1

It has been shown that there is a direct relation between the AgNO_3_ concentration and antibacterial properties of the synthesized nanocomposites. As the Ag content increases, the antibacterial property also increases. Smaller size of the nanoparticles makes more impact of toxicity on the bacteria, due to the greater extend of adsorption at the surface.^121^

**Fig. 9:**
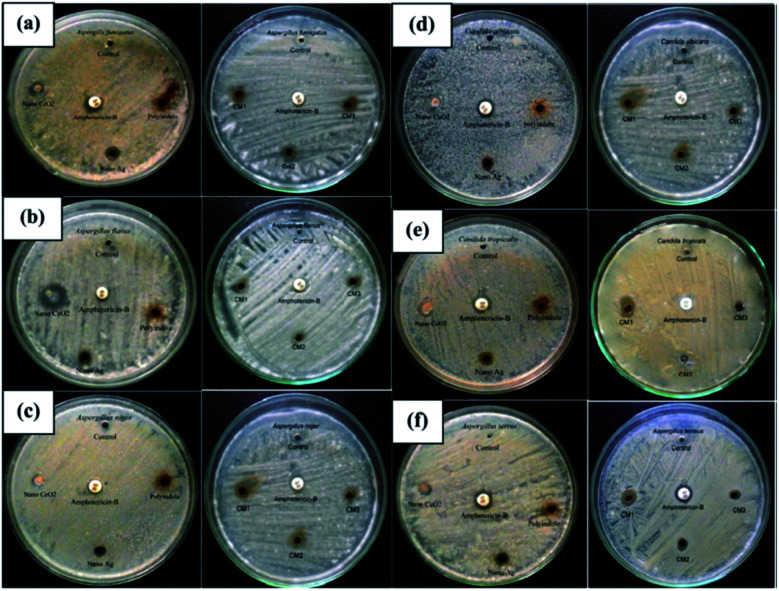
Antifungal response of polyindole/Ag–CeO_2_ systems and individual constituents against (a) *A. fumigates*, (b) *A. flavus*, (c) *A. niger*, (d) *C. albicans*, (e) *A. terreus* and (f) *C. tropicalis*. Reprinted with permission from ref. 120; copyright © Elsevier.

The antifungal activity evaluation of the systems was carried out by agar well diffusion method against the pathogenic fungal species such as *Aspergillus fumigatus*, *Aspergillus flavus*, *Aspergillus niger*, *Candida albicans*, *Aspergillus terreus*, and *Candida tropicalis*. Fig. 9 shows the zone of inhibition against the selected pathogens.

It has been observed that, the polyindole does not exhibit any activity against the selected fungi. Also, the anti-fungal activity does not increase in the presence of Ag nanoparticles. The nanocomposites exhibit moderate antibacterial and minimum antifungal activities against the selected pathogens.

While comparing the antibacterial activity of the nanocomposites with its constituents, it has been found that the nanocomposites exhibited better antibacterial response against the pathogens. The same trend was observed for antifungal activity as well. This might be due to the synergistic enhancement in properties of the partners. From the above results, the polyindole/Ag–CeO_2_ nanocomposite proved to be an efficient antimicrobial agent.

Cobalt oxide nanoparticles are found to be well known antibacterial agent.^122^ In an interesting article, Ag nanoparticles, Co_3_O_4_ nanoparticles and Ag/Co_3_O_4_ nanocomposites of different weight ratio were synthesized *via* an environmental friendly, economical and green synthetic strategy.^123^ They were then subjected to antimicrobial activity evaluation against pathogenic microorganisms which include Gram-negative bacteria (*Escherichia coli* and *Salmonella*) as well as Gram-positive bacteria (*Marsa*, *Listeria*, *Staphylococcus aureus*, *Bacillus subtilis*) and also a pathogenic fungal species, *Candidia*. The results revealed that the systems displayed inhibition against the tested pathogens.

**Fig. 10:**
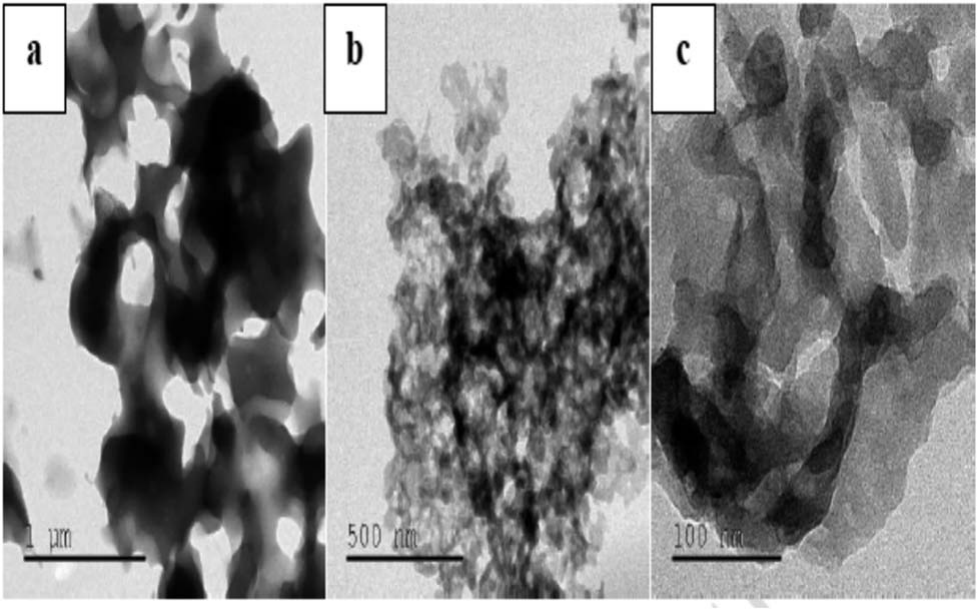
TEM images of polyindole at different magnifications. Reprinted with permission from ref. 124; copyright © Elsevier.

M. Elango *et al.* carried out a systematic study on the development, characterization and antimicrobial potency investigation of polyindole stabilized Ag–Co_3_O_4_ nanocomposites.^124^ The crystallinity was found to be increasing, with an increase in Ag content. XRD results show that the polyindole acts as a reducing as well as a stabilising agent for AgNO_3_ to develop polyindole/Ag–Co_3_O_4_ systems. Porous structure of the polyindole, as evident from TEM images (Fig. 10), makes effective incorporation of Ag and Co_3_O_4_ into it.

The developed polyindole/Ag–Co_3_O_4_ nanocomposites were subjected to antibacterial and antifungal activity by disk diffusion method. Bacterial species selected for the studies were *B. subtilis*, *S. aureus*, *S. pneumoniae*, *E. coli*, *P. vulgaris* and *K. pneumoniae*. The fungal species used were *A. fumigates*, *A. flavus*, *A. niger*, *C. albicans*, *A. terreus* and *C. tropicalis*. Ciprofloxacin and amphotericin-B were used as the reference antibacterial and antifungal agents and a comparison of antimicrobial responses of the nanocomposites with these standards were made. It was a notable observation that the antimicrobial efficiency did not increase with an increase in Ag content.

**Fig. 11:**
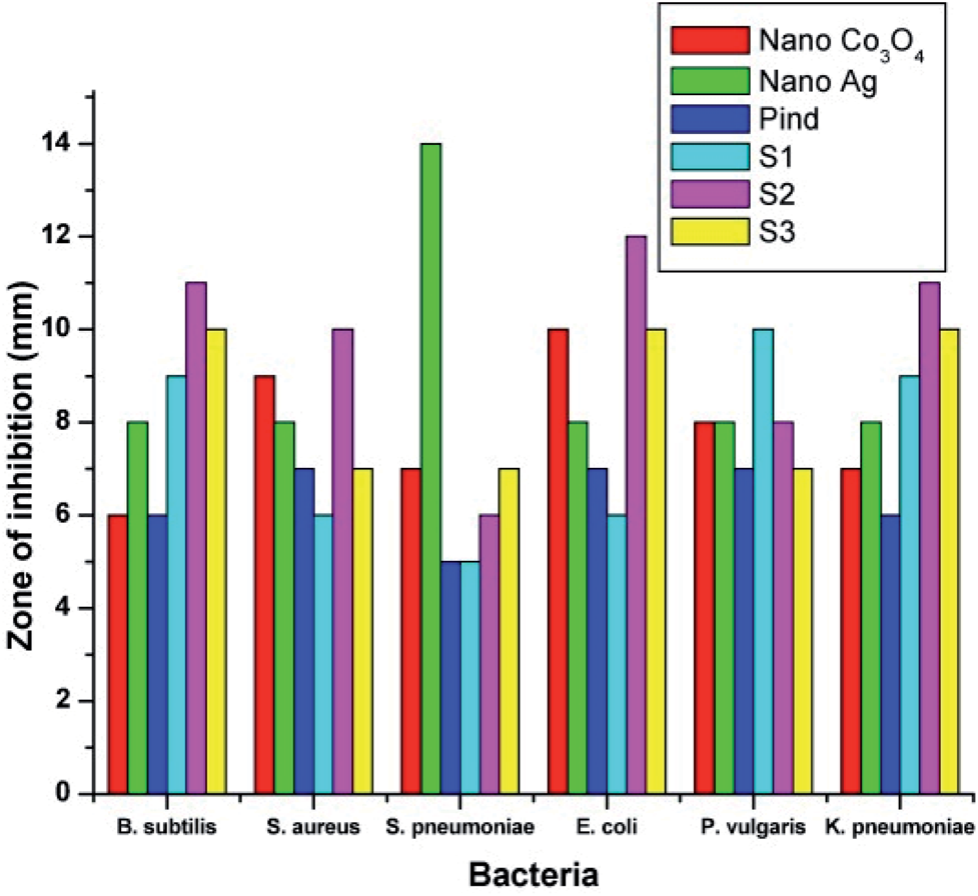
Comparative antibacterial activity of the systems against the pathogens by well diffusion method. Reprinted with permission from ref. 124; copyright © Elsevier.

**Fig. 12:**
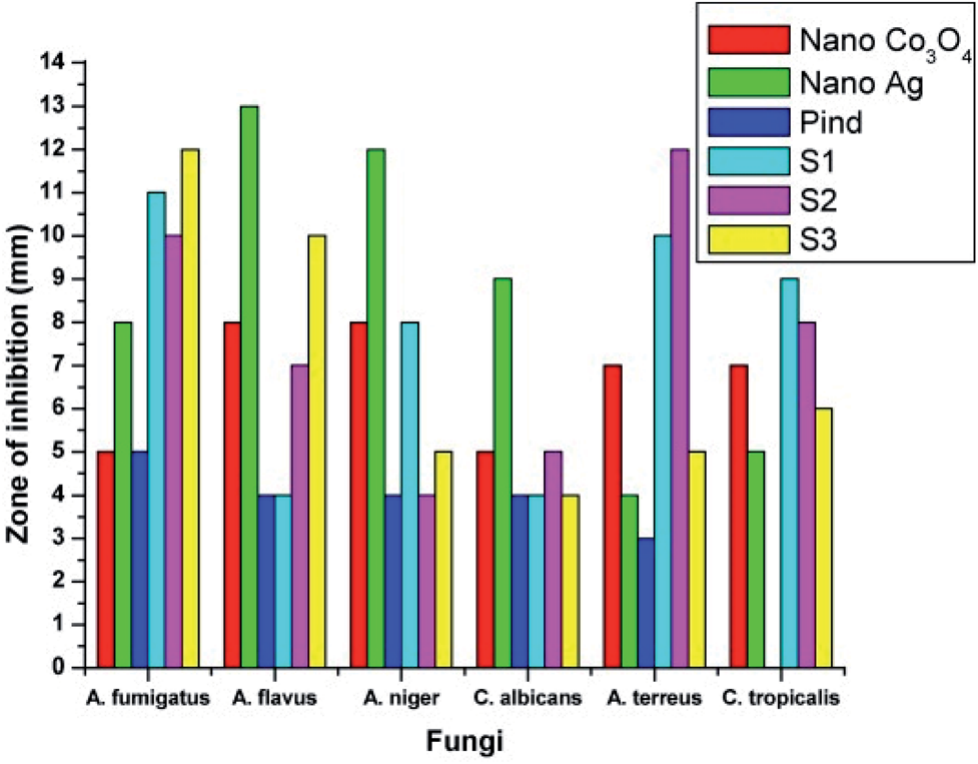
Comparative antifungal activity of the systems against the pathogens by well diffusion method. Reprinted with permission from ref. 124; copyright © Elsevier.

Fig. 11 and 12 shows the comparative antibacterial and antifungal activity of the polyindole/Ag–Co_3_O_4_ systems, respectively. The polyindole/Ag–Co_3_O_4_ nanocomposites exhibited better antibacterial activity than its constituents. The antibacterial activity is due to the interaction between the nanocomposite surface and bacterial cell wall. As the size of the composite particles remains smaller, they can simply pierce the cell wall of bacteria, causing severe toxicity to the bacterial species.^99^ Conducting polymers have shown to cause cell death of bacteria, owing to their excellent antibacterial properties. The nanocomposites may have the sensitivity towards bacterial and fungal cell wall structures, which contribute to their antibacterial activities.^125–127^ All the developed systems exhibited good antibacterial activity against the selected pathogenic microorganisms. The results facilitate the need for further research in order to explore the applications of polyindole/Ag–Co_3_PO_4_ nanocomposites as efficient biomedical agents.

Solubility product (*K*_sp_) of Ag_3_PO_4_ is 1.4 × 10^−16^ and solubility is 0.02 g per litre at 25 °C, is partially soluble in water. Hence, it can slowly release Ag^+^ as an antibacterial agent.^128^ Simple solution-based precipitation methods may be employed for the controlled synthesis of Ag_3_PO_4_ nano-crystals, having good antibacterial properties.^129^ Their antibacterial activity depends upon the size and smaller crystals exhibit better antibacterial activity, owing to high specific surface areas. Upon irradiating with visible light, the antibacterial properties of Ag_3_PO_4_ could be greatly enhanced even more than commercial streptomycin.^130,131^

A latest study has been devoted for the evaluation of antibacterial, anti-cancerous and anti-inflammatory properties of bioactive Ag_3_PO_4_/polyindole nanocomposites. Ag_3_PO_4_ nanocrystals were grown *in situ* inside polyindole matrix to fabricate bioactive Ag_3_PO_4_/polyindole nanocomposites.^132^ XRD, SEM, TEM, EDX and Diffused Reflectance Spectroscopy techniques were used for the characterization of the synthesized samples. The antibacterial, anticancer and anti-inflammatory activity assays proved their outstanding ability to act as a potential biomedical agent. It is notable that even polyindole alone showed antibacterial properties for long time than Ag_3_PO_4_.

**Fig. 13:**
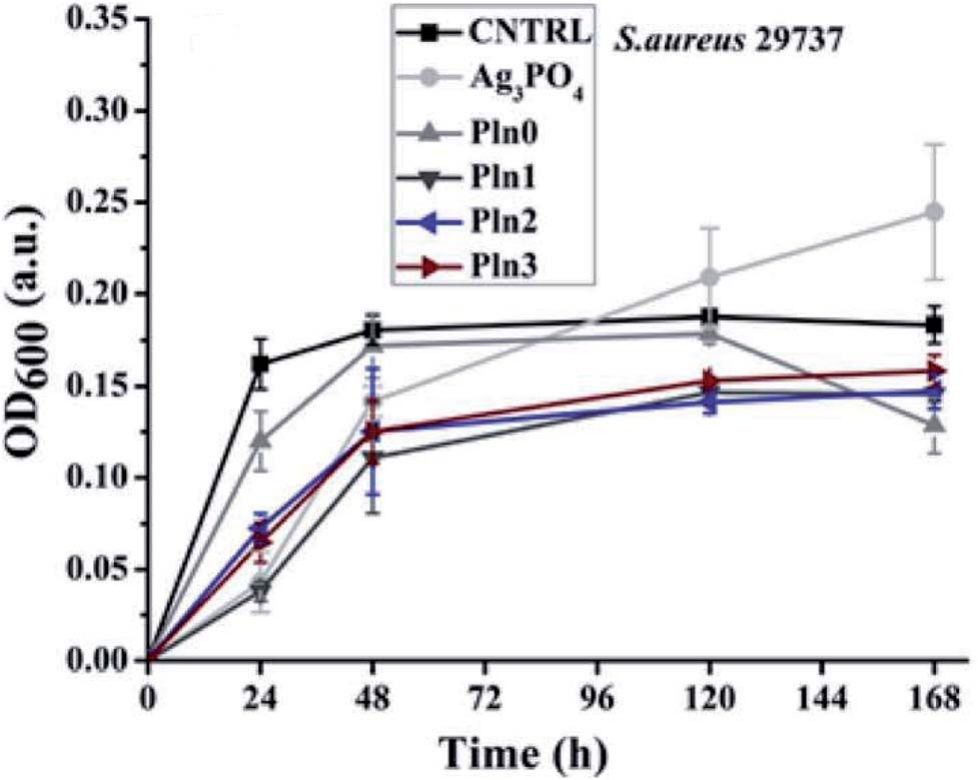
Optical density *versus* time plot of Ag_3_PO_4_/polyindole nanocomposites. Reprinted with permission from ref. 132; copyright © Royal Society of Chemistry.

The intracellular ROS generation accounts for the long-standing antibacterial activity of the nanocomposites. When the composition of the polyindole was 50% of Ag_3_PO_4_, the intracellular ROS generation was greater, for a long time. Minimum inhibitory concentration (MIC) of Ag_3_PO_4_/polyindole has been observed to be equivalent to those of some metallic oxide nanoparticles.^133,134^ It was found that the nanocomposites show inhibition property against the bacterial stains, while virgin polyindole doesn't show any activity at all, this concentration range. This may be owing to the synergistic effect of the partners. Optical density of *E. coli* with virgin polyindole and Ag_3_PO_4_/polyindole nanocomposites was found to follow a similar pattern to that of the control, with lower optical density showing the suppression of bacterial growth and the nanocomposites considerably suppressed *E. coli* to larger level than the virgin polyindole (Fig. 13).

Furthermore, Ag_3_PO_4_ nanocomposites displayed anticancer activity with little toxicity towards other healthy cells. Therefore, more and more research programs have to be carried out to unveil their potential medical applications.

#### 2.2.2. Polyindole/graphene nanocomposites

**Fig. 14:**
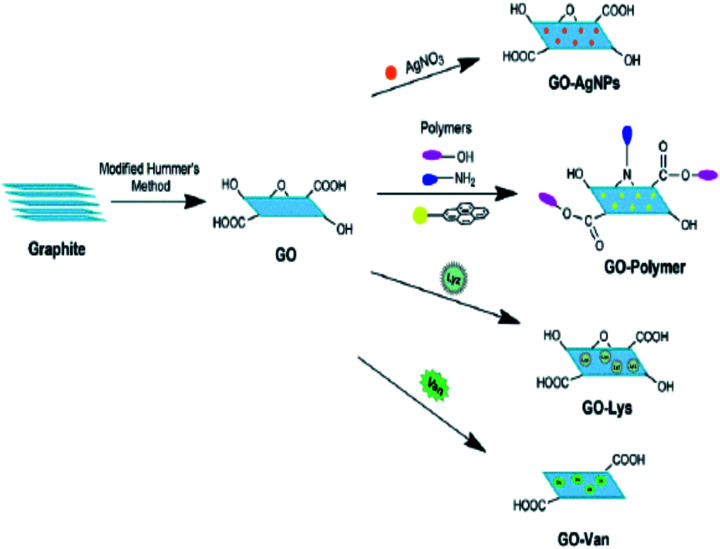
Schematic representation of possibility of functionalisation of graphene, for the development of antibacterial materials. Reprinted with permission from ref. 140; copyright © MDPI.

Graphene and its derivatives could act as potential antimicrobial agents.^135^ Their antimicrobial activity is attributed to the oxidative mechanism which has direct relation with the higher defect density. Their interactions with living cells mainly depend on factors such as degree of hydrophilicity, purity, level of functionalization, lateral size and layer number.^136,137^ However, the biocompatibility studies of graphene and its derivatives show some controversial reports.^138^ They found to exhibit potential cytotoxic and genotoxic effects.^139^ By incorporating graphene and its derivative nanoparticles into biocompatible polymer matrices, their cytotoxic effects can be minimized. Fig. 14 shows a schematic representation of possibility of functionalisation of graphene, for the development of antibacterial materials.^140^

A systematic investigation has been carried out for the *in vitro* and *in vivo* antimicrobial activity evaluation by using polyindole/graphene nanocomposites with methicillin resistant *Staphylococcus aureus* (SA) pathogen. The π–π interaction between the graphene and polyindole dramatically improved the dispersion of graphene in the polyindole matrix. The antibacterial potency of freshly prepared graphene@polyindole nanocomposites with resistant SA isolates have been evaluated.^87^ The standard antibiotic used was vancomycin. The interaction of graphene@polyindole nanocomposite with bacterial cell wall caused its disintegration, which was clearly understood from the electron microscopic studies. Significantly, the graphene@polyindole found to exhibit minimal toxicity to the mammalian cells and hence can effectively eradicate the MRSA strain with appreciable biocompatibility.

**Fig. 15:**
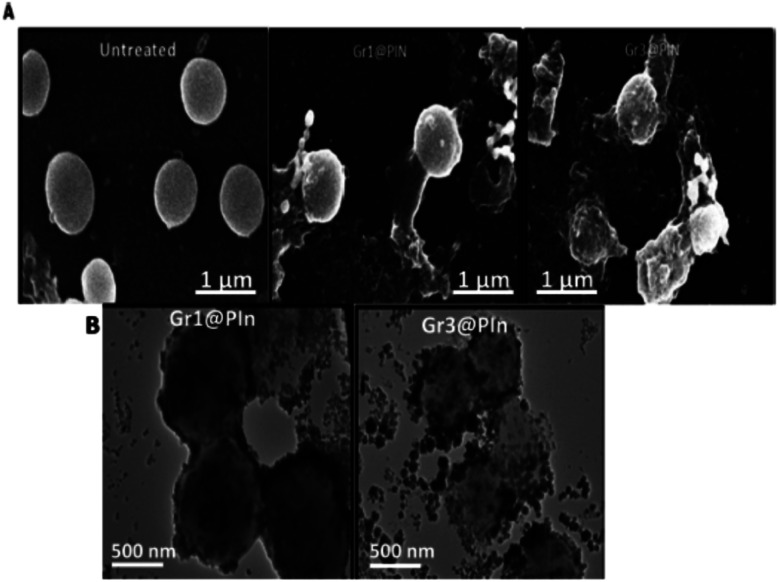
(A) SEM micrographs of the bacteria treated with graphene@polyindole nanocomposites. (B) TEM images indicating the interaction of the nanocomposites with bacterial strains. Reprinted with permission from ref. 87; copyright ©American Chemical Society.

The evaluation of the mechanism of antibacterial property showed that firstly, the graphene@polyindole stick to the bacterial surface, and then it creates an irreversible interruption on the layer of the membrane of the bacteria. After that it eventually penetrated into the cells, and effectively hindered the activity of proteins, leads to bacterial apoptosis *in vitro*. Furthermore, the skin infection mediated by *S. aureus* in BALB/C mice was effectively treated with the synthesized graphene@polyindole nanocomposites. Fig. 15(A) shows the SEM micrographs of the bacteria treated with graphene@polyindole nanocomposites. In the case of untreated samples, the morphology was found to be spherical with smooth cell surfaces. The nanocomposite treated bacteria possesses wrinkled morphology with rough surfaces. Exposure of the bacteria to graphene@polyindole nanocomposites leads to cell lysis followed by the release of cellular components. Fig. 15(B) represent the TEM images indicating the interaction of the nanocomposites with bacterial strains.

**Fig. 16:**
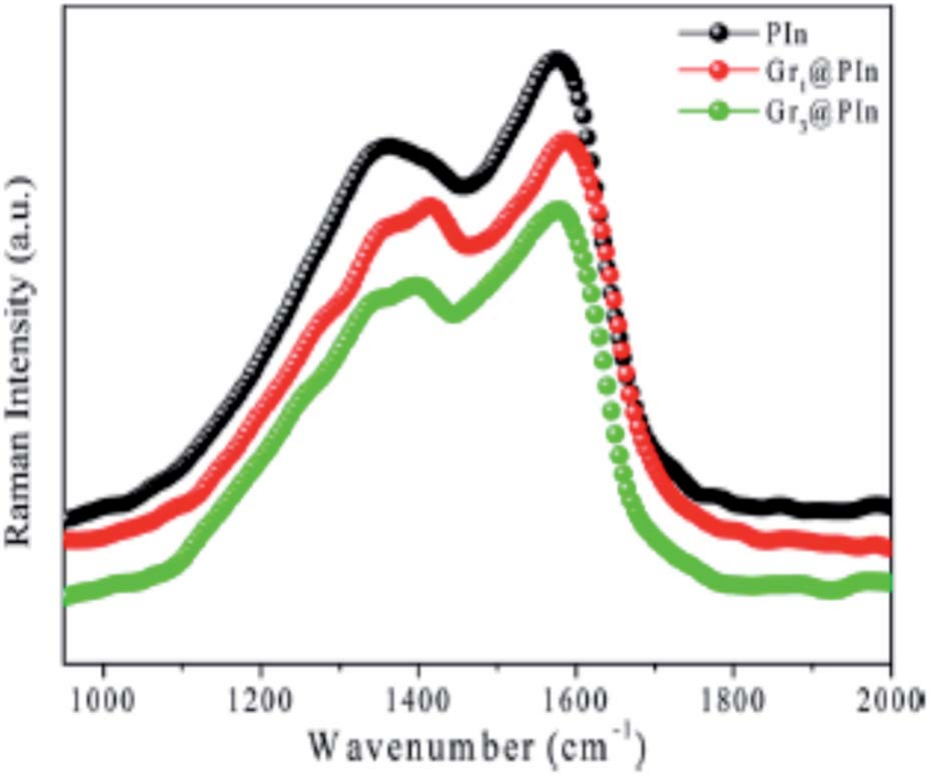
Raman spectrum of polyindole and graphene@polyindole nanocomposites. Reprinted with permission from ref. 87; copyright ©American Chemical Society.

Polyindole-graphene synergy has been further confirmed from Raman spectroscopy as indicated by Fig. 16.

Presence of more sp^3^ carbon atoms, owing to the interaction between polyindole and graphene has been revealed from the intensity ratio of graphene and graphene polymer nanocomposites (Fig. 16). The D band to G band ratio varies from 1.26 to 1.36 for graphene and graphene@polyindole nanocomposites respectively.

**Fig. 17:**
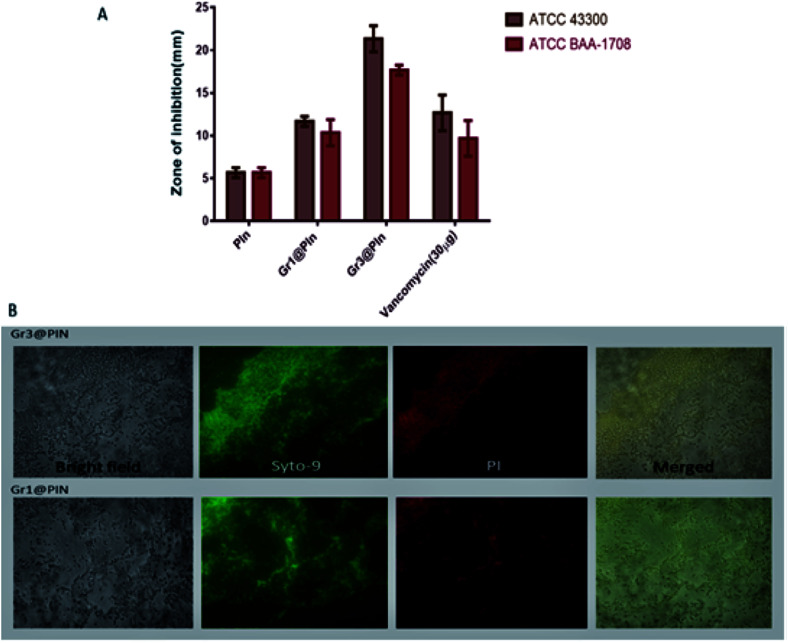
(A) Zone of inhibition against MRSA strains as a measure of anti-microbial activity. (B) The images of fluorescence of MRSA ATCC BAA-1708 were taken by a microscope after treating the cells using different graphene@polyindole formulations. Reprinted with permission from ref. 87; copyright ©American Chemical Society.

The antibacterial potency of the systems, evaluated by agar diffusion assay has been shown as Fig. 17(A). Fluorescence microscopic techniques have been employed to evaluate the live-dead assay, against MRSA stains and represented as Fig. 17(B). Green fluorescence has been observed for control groups, while the samples treated for 3 hours appears to be red in colour, owing to the dye binding with bacterial DNA. The study showed that the nanocomposite is very effective for inhibition of the *S. aureus*-facilitated RBCs lysis. The work highlights the possibilities of future research for the development of a biocompatible and efficient biomedical agent against methicillin resistant *Staphylococcus aureus* (SA) pathogen.

#### 2.2.3. Polyindole/ZrO_2_ nanocomposites

Zirconia nanoparticles and mixed ligand complexes of zirconium were evaluated for their antibacterial efficiency against bacterial strains – *E. coli*, *S. aureus* and fungal strain – *A. niger*.^141^ It was a noteworthy observation that ZrO_2_ nanoparticles and Zr(iv) complexes exhibited crystal plane-dependent interaction with the micro-organisms.^141^ The study could not explain how the killing capacity of these nanoparticles is related their active surface area. They could provide an estimation of crystal plane-dependent bacterial activity of nano ZrO_2_ and their mixed ligand complexes.

Another group of researchers assessed nano zirconia for their antimicrobial activity *via* well disc diffusion method.^142^*B. subtilis* and *S. aureus* (Gram positive) and *E. coli* and *P. aeruginosa* (Gram negative) were selected as reference bacterial strains. Since *P. aeruginosa* possess a negatively charged cell surface, nano zirconia shows an efficient inhibitory action at higher concentrations. The *in vitro* and *in vivo* experiments reveal the possibilities of exploiting the biomedical applications of ZrO_2_ nanoparticles.

**Fig. 18:**
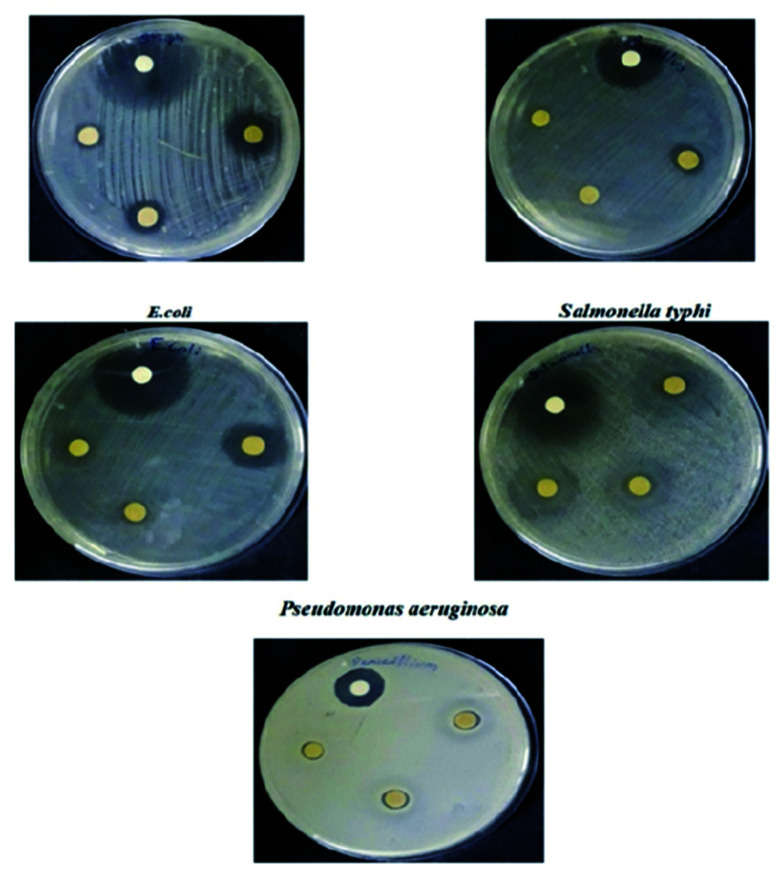
Antibacterial activity of polyindole/ZrO_2_ systems against selected bacterial strains as a measurement of zone of inhibition. Reprinted with permission from ref. 143; copyright © Elsevier.

S. Anandhi *et al.* synthesized polyindole/ZrO_2_ nanocomposites, by using mixing solution method.^143^ SEM analysis was used to understand the morphology of the synthesized polyindole, nano ZrO_2_ and polyindole/ZrO_2_ nanocomposites. FTIR, UV-Visible and NMR techniques have been employed for the structural confirmation of the synthesized nanocomposites. The degree of crystallinity and crystalline sizes were determined from XRD analysis. The thermal stability of the synthesized composites were analysed from TGA and DSC studies. EDAX technique was used to demonstrate elemental analysis and chemical characterization. The antibacterial activity of the synthesized nanocomposites were carried out on five microorganisms – *Staphylococcus aureus*, *Bacillus subtilis*, *Pseudomonas aeruginosa*, *Salmonella typhi* and *E. coli*. Table 6 presents the antibacterial results of the polyindole/ZrO_2_ systems, and zone of inhibition have been represented as Fig. 18.

**Table 6:** Antibacterial activity of polyindole/ZrO_2_ systems. Reprinted with permission from ref. 143; copyright ©Elsevier

Microbes	Gram staining	Inhibition zone (mm)			Antibiotic (1 mg mL^−1^)
		Concentration of the samples (1 mg mL^−1^)			
*Staphylococcus aureus*	Gram positive	20	13	10	35
*Bacillus subtilis*		10	8	8	20
*E. coli*	Gram negative	15	10	9	26
*Salmonella typhi*		13	10	8	30
*Pseudomonas aeruginosa*		15	10	7	30

**Fig. 19:**
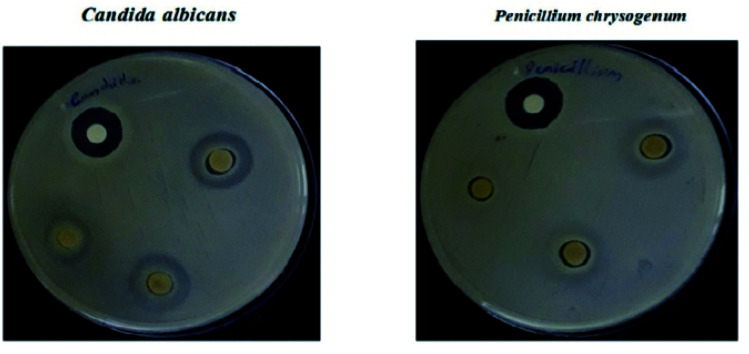
Antifungal activity of the composite material against selected bacterial strains as a measurement of zone of inhibition. Reprinted with permission from ref. 143; copyright © Elsevier.

The polyindole/ZrO_2_ systems were also subjected to antifungal activity studies against pathogenic fungal strains – *Candida albicans* and *Penicillium chrysogenum*. The results were compared with the standard antibiotic – amphotericin-B. The synthesized polyindole/ZrO_2_ nanocomposites displayed excellent antifungal properties. Table 7 shows the antifungal data of the systems, and zone of inhibition have been represented as Fig. 19.

**Table 7:** Antifungal activity of polyindole/ZrO_2_ systems. Reprinted with permission from ref. 143; copyright © Elsevier

Microbes	Zone of inhibition (mm)			Antibiotic (1 mg mL^−1^)
	Sample concentration (1 mg mL^−1^)			
	1000	750	500	
*Candida albicans*	16	15	14	17
*Penicillium chrysogenum*	11	10	8	18

#### 2.2.4. Polyindole/TiO_2_ nanocomposites

The enhanced antimicrobial activity and biocompatibility of nano-TiO_2_ coatings were comprehensively explored.^144,145^ Unique nano-TiO_2_ coatings on Ti substrates, introduced *via* temperature-controlled atomic layer deposition (ALD), displayed excellent activity against *Staphylococcus aureus*, *Escherichia coli* and methicillin-resistant *Staphylococcus aureus*.

**Fig. 20:**
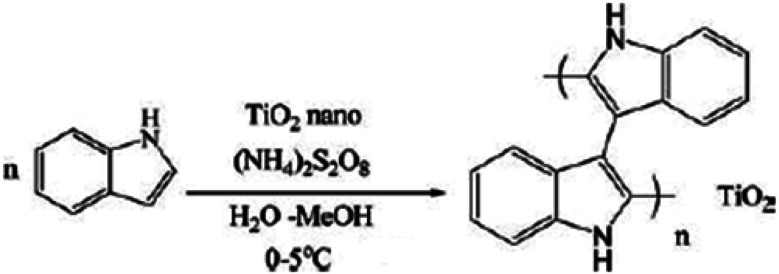
Synthesis strategy of polyindole/TiO_2_ nanocomposites. Reprinted with permission from ref. 146; copyright © Taylor and Francis Ltd.

A systematic investigation was conducted on the development and antibacterial activity evaluation of polyindole/TiO_2_ nanocomposites.^146^ They employed ultrasound condition by aqueous *in situ* polymerization method. A representation of the synthesis strategy has been shown as Fig. 20. FTIR, UV-visible, XRD, TGA and SEM techniques were used for the structural characterization of the synthesized nanocomposite samples.

**Fig. 21:**
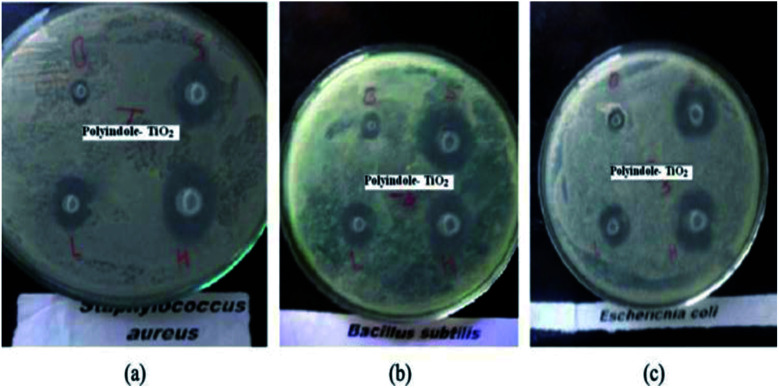
Antibacterial activity as a measure of zone of inhibition for polyindole/TiO_2_ nanocomposite against (a) *Staphylococcus aureus*, (b) *Bacillus subtilis* and (c) *E. coli*. Reprinted with permission from ref. 146; copyright © Taylor and Francis Ltd.

The XRD pattern indicated that TiO_2_ is in the anatase form. A significant antibacterial activity could be observed for the nanocomposite samples against *B. subtilis* and *S. aureus* (Gram-positive). However, a moderate activity was observed against *E. coli* (Gram-negative) whereas no activity was observed against *S. typhi*. The zone of inhibition has been indicated in Fig. 21.

It was proposed that the antibacterial activity of the polyindole/TiO_2_ nanocomposite materials is attributed to their ability to inhibit nucleic acids, thiol groups and essential enzymes present on the bacterial cell membranes. Hence, these materials act as a promising candidate and their use could be a novel approach to fight drug-resistant bacterial infections.

#### 2.2.5. Polyindole/nickel–zinc oxide nanocomposites

The antifungal properties of polyindole based nickel–zinc oxide (PIN/Ni–ZnO) nanocomposites was reported recently.^147^ The well-known co-precipitation method, by using a capping agent was selected for the preparation of nickel–zinc oxide nanocomposites. The chemical oxidation method has been employed for the preparation of PIN/Ni–ZnO nanocomposites. The antifungal investigation of PIN/Ni–ZnO nanocomposite and its counterparts have been carried out by using test fungi species *Penicillium chrysogenum*, showed that the nanocomposite has an antifungal activity of 1 cm, the nickel–zinc oxide component showed an activity of 0.7 cm and whereas the nickel oxide, zinc oxide and polyindole were showing no remarkable activity.

From this study, it is evident that when NiO/ZnO nanocomposite combines with polyindole matrix, there observed a higher antifungal activity than the single NiO/ZnO nanocomposites. This enhanced antimicrobial activity of the polymer nanocomposite has been due to the synergistic effect of both the polymer matrix and metal oxide nanoparticle counterparts in favour of appreciable ROS generation. From the above results, we can conclude that the synthesized polyindole/NiO–ZnO nanocomposite could act as a potential antifungal material to fight the fungal infection caused by *Penicillium chrysogenum*.

## 3. Future perspectives

Polyindole based nanocomposites were observed to be a potential biomaterial against the multi-drug resistant microbes. They can be used as an alternative to antibiotics and antifungal drugs. Clinical and *in vivo* applications are largely based on the size of the released nanoparticles from the polymer nanocomposites. Hence, it necessitates the need for effective toxicological studies and investigations of the polymer nanocomposites before initiating clinical trials. Another concern is at the development of an economical as well as an ecofriendly method for the synthesis of polyindole based nanocomposite is required. However, nowadays more and more researchers are coming up with some innovative green synthetic strategies, which may give progress to the field of polyindole based nanocomposites for biomedical applications.

## Conflicts of interest

There are no conflicts to declare.

## Supplementary Material
